# Research trends of exercise therapy of college students in depression from 2002 to 2022: a bibliometric analysis

**DOI:** 10.3389/fnins.2023.1188341

**Published:** 2023-05-12

**Authors:** Zhongzhu Ai, Dongfeng Yuan, Yitong Meng, Zhuo Ai, Sisi Zhu

**Affiliations:** ^1^Faculty of Pharmacy, Hubei University of Chinese Medicine, Wuhan, China; ^2^Key Laboratory of Traditional Chinese Medicine Resources and Chemistry of Hubei Province, Wuhan, China; ^3^Modern Engineering Research Center of Traditional Chinese Medicine and Ethnic Medicine of Hubei Province, Wuhan, China; ^4^College of Art Design, Hubei University of Economics, Wuhan, China; ^5^Department of Student Affairs Management, Hubei University of Chinese Medicine, Wuhan, China

**Keywords:** depression, college students, exercise therapy, bibliometric analysis, research hotspots, VOSViewer

## Abstract

**Background:**

Depression is a serious psychological disorder that college students are experiencing. College students’ depression problems, which can be caused by various factors, have been easily ignored and untreated. In recent years, exercise, as a low-cost and easily accessible method for treating depression, has attracted widespread attention. The purpose of this study is to use bibliometrics to explore the hotspots and trends in the field of exercise therapy of college students in depression from 2002 to 2022.

**Methods:**

We retrieved relevant literature from the Web of Science (WoS), PubMed, and Scopus databases, and generated a ranking table to describe the core productivity in the field. We used VOSViewer software to generate network maps of authors, countries, co-cited journals, and co-occurring keywords to help us better understand the scientific collaboration patterns, potential disciplinary foundations, as well as research hotspots and trends in this field.

**Results:**

From 2002 to 2022, a total of 1,397 articles related to exercise therapy of college students in depression were selected. The key findings of this study are as follows: (1) the number of publications has gradually increased, especially after 2019; (2) United States and its affiliated higher education institutions have made significant contributions to the development of this field; (3) there are multiple research groups in this field, but their connections are relatively limited; (4) the field is relatively interdisciplinary, primarily a convergence of behavioral science, public health, and psychology; (5) based on co-occurring keyword analysis, six main themes were summarized: health-promoting factors, body image, negative behaviors, increased stress, depression coping strategies, and diet.

**Conclusion:**

Our study illustrates the research hotspots and trends for the research of exercise therapy of college students in depression, presents some challenges and new insights, and provides valuable information for further research.

## Introduction

The period of university is a significant time in one’s growth, and the enrollment in university marks that young people are about to enter a transitional period, that is, from adolescence to adulthood. However, during this transition, students are faced with many new challenges, such as learning environment changes, making independent decisions and choices regarding their own lives, and interacting with students from all over the country. Additionally, many students are away from home and their families for the first time, living independently in an unfamiliar city (Cleary et al., 2011). Freshmen, in particular, are more vulnerable and face more challenges in successfully adapting, with psychological health problems commonly persistent and having a negative impact on academic performance and overall wellbeing (Duffy et al., 2020). Facing various challenges in university life, students experience significant pressure, with a larger cross-national study indicating that most students are under stress from at least one of the following: financial situation, health, romantic relationships, relationships with family, work/school relationship, or relationship with a partner (Karyotaki et al., 2020). Prolonged exposure to high levels of stress increases the risk of psychological health problems among university students (Slimmen et al., 2022).

Depression is a condition in which a person feels discomfort with their emotions and cannot change it through self-regulation. Its basic characteristics include feelings of sadness, lack of interest, sluggish thinking, slow reaction, and unwillingness to communicate with friends and family. In severe cases, it can produce feelings of pessimism, despair, pain, and even death. In 2008, the World Health Organization listed major depression as the third leading cause of global disease burden and predicted that it will rank first by 2030 (Malhi and Mann, 2018). Continuous life stress increases the incidence of depression, and patients with major depressive disorder (MDD) generally have anxiety, which makes it more difficult to treat than depression without anxiety. The severity of current anxiety symptoms during a depressive episode is closely related to the duration of subsequent depressive symptoms, and this relationship is stable over decades (Coryell et al., 2012; Fried et al., 2015; Baldwin et al., 2016). In the past two decades, research on depression, anxiety, and other mental health problems among college students has increased exponentially, which may be caused by the following reasons. Firstly, mental health problems among university students around the world have become increasingly severe in recent years, and students’ desire for mental health assistance is stronger (Seyfi et al., 2013; Olfson et al., 2016; Seppala et al., 2020). However, most students have misunderstandings and lack of trust in campus mental health counseling, the stigma of mental illness, difficulty accessing mental health services, doubts about the effectiveness of treatment, and a general lack of urgency (Eisenberg et al., 2011; Ning et al., 2022), which are the main obstacles to college students’ seeking mental health counseling. Secondly, students’ psychological distress is closely related to short-term adverse psychological reactions, such as students’ age, peer relationships, economic independence, academic stress, and learning field (Vazquez et al., 2012). The prolonged psychological stress has led to an increase in the proportion of psychological symptoms such as depression, anxiety, psychological tension, and decreased attention, as well as a higher co-occurrence rate of different psychological symptoms, which is more common among female students and older students (Oksanen et al., 2017). Thirdly, international estimates indicate that approximately one-third of students experience common mental disorder symptoms during their university years, many of whom are in late adolescence, which is a high-risk period for mental disorders. Universities as learning and educational institutions provide more opportunities for improving the mental health of young people (Osborn et al., 2022). Despite the progress of society and the expansion of universities, which has enabled more students to enter universities for learning and living, the provision of psychological support services for students has not developed at an equal pace (Dobson et al., 2012). Most students with depression, anxiety, and other mental health problems have not received effective treatment (Bernhardsdottir and Vilhjalmsson, 2013; Busby et al., 2021).

For the treatment of depression, drugs are undoubtedly a basic way of clinical treatment. However, the potential adverse effects of antidepressant drugs, such as gastrointestinal symptoms, respiratory distress, hepatotoxicity and anaphylaxis, etc., can reduce the treatment compliance and cause inconvenience to patients (Carvalho et al., 2016; Morris et al., 2021). Increasing evidence suggests that non-pharmacological treatments for depression can significantly improve patients’ depressive symptoms and enhance the patients’ emotional regulation abilities (Crowe et al., 2015; Zhang M. et al., 2022). In recent years, physical activity as a low-cost and easily accessible method for treating depression has garnered attention, and several studies have demonstrated its efficacy in reducing depression symptoms, improving physical function such as cardiovascular and cognitive abilities (Majumder et al., 2015; Rahman et al., 2018). A study by Zhu et al. (2020) found that low-intensity physical exercise can significantly improve the psychological health of university students, which may be due to the activation of potential targets in the brain (Gultyaeva et al., 2019). Although some qualitative reviews have summarized the impact of exercise on university students’ depression symptoms, the distribution and contribution of the studies in this field have not yet been analyzed comprehensively using bibliometric methods, and the theoretical core and structure of the field have not been demonstrated (Stanton and Reaburn, 2014; Qi et al., 2022). Hosseini et al. (2018) pointed out that qualitative reviews may have some subjective potential biases. Therefore, it is necessary to systematically evaluate the exercise therapy of college students in depression using quantitative methods.

Bibliometrics analysis provides an objective and systematic approach to discover knowledge structure patterns in the field structure, reveal its disciplinary foundations, identify emerging themes and trends, expose existing problems and challenges in the field, and ultimately contribute to the advancement of the field (He J.B. et al., 2022). This study aims to provide a bird’s-eye view of nearly two decades of research conducted in this area through a bibliometric approach to expand understanding in the fields of exercise therapy of college students in depression. To this end, several commonly used bibliometric indicators are employed in this study, based on the publication quantity trend over time to explore the development trajectory of the field over the past two decades; based on the publication output and co-citation relationship of authors and countries, to explore the key participants promoting research on exercise therapy of college students in depression and potential scientific collaboration patterns in the field; through a co-citation analysis of journals, to explore the potential disciplinary foundations of the field over the past two decades; through a co-occurrence analysis of keywords, to explore the repeatedly discussed research themes and their changing trends over time. Finally, through a comprehensive analysis of all the results, the current research hotspots, status, and future challenges of the field are summarized, and the strengths and limitations of this study as well as future research directions in the field are pointed out. This study will be helpful in guiding policies, research, and practices of higher education institutions and provide some theoretical support for further exploration of the field.

## Methods

### Data collection and search strategy

In this study, documents were collected from three databases: Web of Science (WoS), PubMed and Scopus. The WoS, one of the most standard and widely used databases in bibliometric analysis (Li et al., 2022). Four high-quality indexes in WoS were included in our search scope, namely: The Science Citation Index-Expanded (SCI -Expanded); the Social Sciences Citation Index (SSCI); the Arts & Humanities Citation Index (A&HCI); and the Emerging Sources Citation Index (ESCI). To create a comprehensive literature set on exercise therapy of college students in depression, retrieval was performed from three aspects. Firstly, considering the impact of factors such as stress, anxiety, and mental illness on depression symptoms, the WoS search formula was set as follows: [“depression” OR “stress” OR “anxiety” OR “anxious” OR “mental illness” OR “mental disorder*” OR “depressed” OR “psychological distress” OR “psychopathology”]. Secondly, to limit the research objects to college students, the WoS search formula was set as follows: [“university student*” OR “college student*” OR “higher education” OR “undergrad student” OR “master’s student” OR “postsecondary education” OR “undergraduate*” OR “tertiary education” OR “postsecondary education” OR “doctoral student” OR “Ph.D. student”]. Thirdly, to obtain the impact of exercise on depression symptoms, the WoS search formula was set as follows: [“exercise” OR “physical training” OR “exercise therapy” OR “exercise treatment” OR “sport* therapy” OR “sport* intervention” OR “exercise prevention*” OR “physical activity”].

PubMed, a crucial search engine, was selected as one of the search engines for this study, as its main source, MEDLINE, is focused on the medical field. The aim of this study was to collect a dataset of literature on exercise therapy of college students in depression, so PubMed was selected as one of the search engines for this study. The search strategy on PubMed platform was set as follows: [((((((“Depression”[Majr]) OR “Anxiety”[Majr]) OR “Mental Disorders”[Majr]) OR “Psychological Distress”[Majr]) OR “Stress Disorders”[Majr]) AND (((((university students[Title/Abstract]) OR (college students[Title/Abstract])) OR (undergrad students[Title/Abstract])) OR (master’s students[Title/Abstract])) OR (doctoral student[Title/Abstract]))) AND (((exercise[MeSH Major Topic]) OR (sports therapy[Title/Abstract])) OR (physical activity[Title/Abstract]))].

Scopus is one of the largest citation bases, with extensive and comprehensive coverage. Literature in the this database was searched as follows: ((TITLE-ABS-KEY (depression) OR TITLE-ABS-KEY (stress) OR TITLE-ABS-KEY (“anxiety”) OR TITLE-ABS-KEY (“mental illness”) OR TITLE-ABS-KEY (“mental disorder”)) AND (TITLE-ABS-KEY (“college student”) OR TITLE-ABS-KEY (“university student”) OR TITLE-ABS-KEY (“undergrad student”) OR TITLE-ABS-KEY (“postsecondary education”) OR TITLE-ABS-KEY (“doctoral student”)) AND (TITLE-ABS-KEY (exercise) OR TITLE-ABS-KEY (“exercise therapy”) OR TITLE-ABS-KEY (“exercise treatment”) OR TITLE-ABS-KEY (“exercise treatment”) OR TITLE-ABS-KEY (“physical activity”))).

### Data screening and extraction

**Figure 1:**
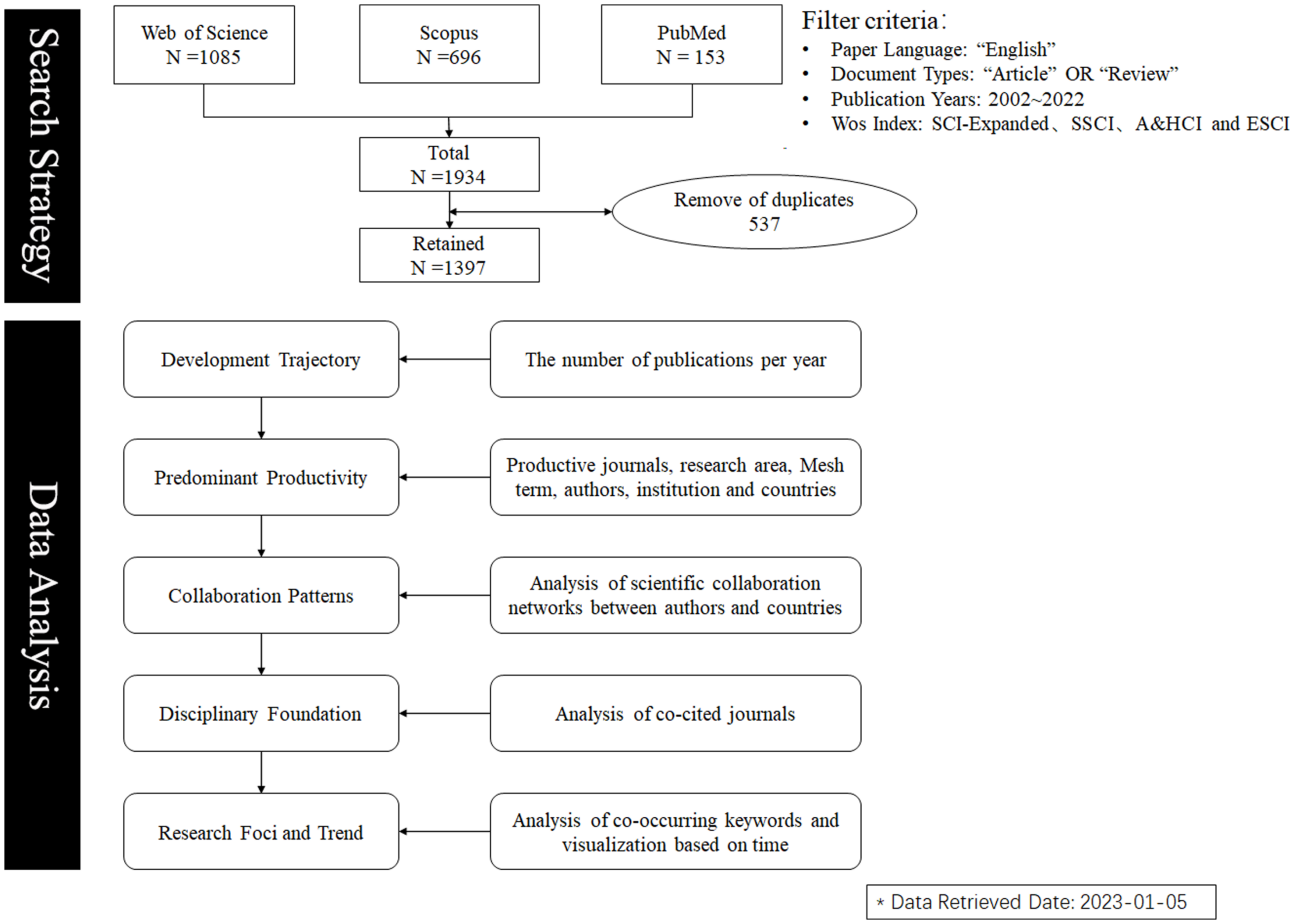
Search strategy and research framework.

The inclusion and exclusion criteria were as follows: The document type was set to “Article” and “Review,” the time period was set from January 2002 to December 2022, and the language was limited to English. In addition, during the entire process of data retrieval and selection, two reviewers (DY and YM) independently and sequentially screened potential studies identified by the search strategy based on inclusion and exclusion criteria, by reading titles, abstracts and full-text articles. Discrepancies were discussed, and a consensus was reached. After screening, a total of 1,085 articles were included in WoS, 153 articles were included in PubMed, and 696 articles were included in Scopus. After merging and removing 537 duplicate articles by Endnote 9 software, a total of 1,397 articles were finally obtained. The article retrieval and data extraction were completed within 1 day on January 5, 2023, to avoid potential biases caused by daily database updates. The process of data collection and analysis approach is shown in Figure 1.

### Data analysis tool and methods

Bibliometrics analysis encompasses the exploration of distribution of publication and research topics, as well as citation counts. Descriptive statistics and bibliometric methods are utilized to analyze the literature database, providing a general picture of the evolution and current status of research in the area of exercise therapy of college students in depression. The number of publications per year is counted to describe the development trajectory of this field in the past two decades. A ranking table is compiled based on publication output of core journals, researcher area, Mesh term, authors, institutions, and countries that make significant contributions to the development of this field, to describe its core productivity. Furthermore, Citations，Average Citations and H-index corresponding to these elements were collected to assess their influence in the field (Li et al., 2021; He T. et al., 2022), and the Journal Impact Factor (IF) and the Journal Citation Reports (JCR) of 2021 for the top journals were also collected in this study.

A bibliometric analysis of the publication database was conducted using VOSViewer software in order to explore and visually represent the scientific collaboration, potential disciplinary foundations, and research foci and trends in the field. VOSViewer is a free computational software for viewing and constructing bibliographic maps. In VOSViewer, the units of analysis include journals, publications, citations, authors or countries, depending on the focus of the analysis and the type of database. Circular nodes in the map represent the units of analysis, the size of the nodes represents their proportion, and their positions reflect the similarity between different nodes in the map, with more similar nodes being closer in distance. The lines connecting different nodes represent the relationships between the nodes, with the thickness of the line representing the strength of the relationship; the color of the node represents different clusters, which are divided based on the degree of relatedness between the nodes (Van Eck and Waltman, 2014). The construction of the network map in VOSViewer is based on a distance-based approach, which consists of three steps. First, the software standardizes the processing of each node in order to eliminate differences between the nodes. Second, the software constructs a two-dimensional map, where the distance between nodes represents the similarity between these nodes. In the third step, VOSViewer categorizes closely related nodes into the same cluster in order to achieve the goal of clustering (Van Eck et al., 2010).

In this study, we first conducted a collaboration analysis between authors and countries to explore the scientific collaboration network and social relationships in the field of exercise therapy of college students in depression. In this analysis, each node in the map represents an author or country, the connections between different nodes reflect their relationships, clusters represent scientific collaboration networks, which can be interpreted as groups of authors or countries who often publish papers together, and the thickness of the connections reflects the closeness of their collaboration. Secondly, to explore the potential disciplinary foundation in this field, we adopted the co-citation analysis method of journals, where the analysis object is the journals in the database, and the map reflects the strength of the co-citation relationship. Co-cited journals refer to two journals that are cited by the same third journal, the more times a pair of journals are cited by other journals, the stronger their co-citation relationship is. Journals that are often co-cited are considered to have a strong theoretical and semantic foundation (Hernandez-Torrano et al., 2020). Therefore, in this study, clusters of journals that are often jointly cited can be interpreted as the basic disciplines in this field. Finally, through the co-occurrence analysis of keywords, we explore the research foci and trend in this field. In this analysis, the analysis object is the author’s keywords, the higher the frequency of two keywords appearing in the same record, the stronger their co-occurrence relationship is, and the clustering of co-occurrence keywords represents the local hot topics of research on university student depression and sports activities in recent 20 years. Furthermore, we also conducted a temporal co-occurrence analysis based on keywords, where the color legend represents the average year of high-frequency keywords’ appearance (Li and Liu, 2020). This analysis helps us understand the trend of how the hotspots in the field of research change over time.

## Results

### Overall development trajectory

**Figure 2:**
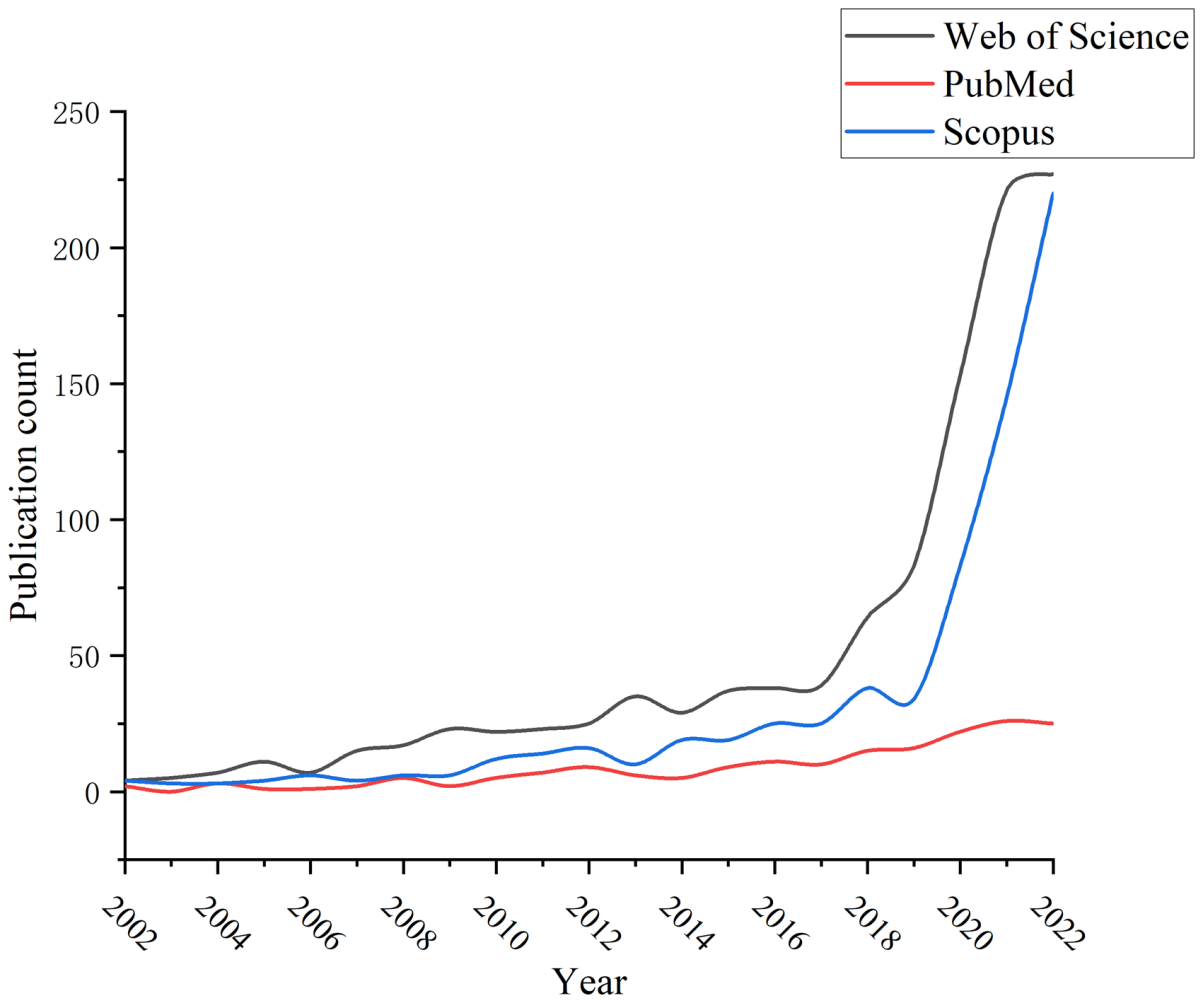
Change in number of publications per year.

The development pattern of a certain field in recent years can be well illustrated by the change in the number of publications per year. Figure 2 shows the development trajectory of publication data from 2002 to 2022 on research on exercise therapy of college students in depression. In general, in the past 20 years, more and more scholars have shown a strong interest in issues such as university students, depression and physical exercise, which can be roughly divided into three stages: the first stage, the germination stage (2002–2010), a small number of articles were found in both the WoS, PubMed, and Scopus databases, with slow increase; the second stage, the growth stage (2010–2019), the number of publications in both databases has been increased and maintained a stable speed; the third stage, the take-off stage (2019–2022), during this period, the annual number of publications in three databases significantly increased, especially after 2019, the number of publications of 2020 in WoS and Scopus was almost twice that of 2019, and it showed a fast growth in the following 2 years.

### Predominant productivity

#### Productive publication source

Overall, 1,085 articles were published by 490 journals in the WoS dataset, 153 articles were published by 99 journals in the PubMed dataset, and 696 articles were published by 158 journals in the Scopus dataset. Table 1 show the sources of publications with higher counts. Obviously, *International Journal of Environmental Research and Public Health* is the most productive publication source in this field, accounting for about 7.47, 10.46, and 7.47% of the three databases respectively, followed by *Journal of American College Health* (WoS, 58; PubMed, 18; Scopus, 40) and *Frontiers in Psychology* (WoS, 37; Scopus, 25). Notably, *PLoS One*, despite being a relatively young and interdisciplinary journal, has also published plenty of research in this field, ranking fourth.

**Table 1:** Top publication sources in the field of exercise therapy of college students in depression.

Rank	Source title	Publication count n (%)			Citations	Average citation	IF (2021)	JCR (2021)
		WoS (*n* = 1,085)	PubMed (*n* = 153)	Scopus (*n* = 696)				
1	International Journal of Environmental Research and Public Health	81 (7.47)	16 (10.46)	52 (7.47)	1,074	13.26	4.614	Q2
2	Journal of American College Health	58 (5.35)	18 (11.77)	40 (5.75)	1,403	24.19	2.394	Q3
3	Frontiers in Psychology	37 (3.41)	__*	25 (3.59)	356	9.62	4.232	Q1
4	Plos One	20 (1.84)	6 (3.92)	12 (1.72)	639	31.95	3.752	Q2
5	Psychology of Sport and Exercise	17 (1.57)	__	6 (0.86)	439	25.82	5.118	Q2
6	BMC Public Health	13 (1.20)	5 (3.27)	8 (11.5)	540	41.54	4.135	Q2
7	Nutrients	13 (1.20)	6 (3.92)	14 (2.01)	113	8.69	6.706	Q1
8	Frontiers in Psychiatry	10 (0.92)	__	12 (1.72)	129	12.90	5.556	Q2
9	Frontiers in Public Health	10 (0.92)	1 (0.65)	7 (1.01)	6	0.60	6.461	Q1
10	International Journal of Mental Health and Addiction	10 (0.92)	__	7 (1.01)	167	16.70	11.555	Q1

*__: no related records.

#### Productive research areas and foci in the past 20 years

Table 2 presents the top several fields with the greatest contributions to research on exercise therapy of college students in depression. Over half of the records in the WoS dataset were published in the area of *Public Environmental Occupational Health* (27.01%) and *Psychology* (24.24%). The field of *Psychiatry* also demonstrated strong influence with 161 studies published and accounting for nearly 15% of the publications. Similarly, the area of *Education and Educational Research* accumulated over 100 publications in the field, representing approximately 10%. Other relevant research in the field was primarily focused in areas such as public environment, sports science, and medicine, including *Environmental Sciences*, *Ecology, Sport Sciences*, *Nutrition and Dietetics*, *General Internal Medicine*, *Health Care Sciences and Services*, and *Neurosciences and Neurology*.

**Table 2:** Top research areas in WoS (*N* = 1,085).

Rank	Research areas	Records of occurrence in publications, n (%)	Citations	Average citation	H index
1	Public Environmental Occupational Health	293 (27.01)	6,705	22.88	42
2	Psychology	263 (24.24)	6,425	24.43	40
3	Psychiatry	161 (14.84)	3,788	23.53	35
4	Education Educational Research	108 (9.95)	2,175	20.14	23
5	Environmental Sciences Ecology	93 (8.57)	1,343	14.44	17
6	Sport Sciences	74 (6.82)	1,206	16.3	21
7	Nutrition Dietetics	66 (6.08)	1805	27.35	24
8	General Internal Medicine	56 (5.16)	828	14.79	14
9	Health Care Sciences Services	51 (4.70)	1,181	23.16	15
9	Neurosciences Neurology	51 (4.70)	1,278	25.06	18

Medical Subject Headings (MeSH) is a medical vocabulary resource created by the National Library of Medicine (NLM). NLM provides biomedical keywords as its MeSH terms for all PubMed literature, and professional researchers label each article with corresponding MeSH terms. As a result, the accuracy and relevance among articles related to MeSH terms are high (Da et al., 2021). Table 3 shows the top 10 MeSH terms that occur frequently in the topic-related articles retrieved in the PubMed dataset. The most frequent term was *Human*, followed by *Female*, *Male*, and *Students*, which were recorded over 100 times and accounted for more than 70%. In addition, other frequently appearing MeSH terms were mainly focused on psychology, university students, epidemiology, and exercise fields, including *Young Adult*, *Psychology*, *Universities*, *Adult*, *Epidemiology*, and *Exercise*.

**Table 3:** Top research foci in PubMed (*N* = 153).

Rank	Mesh term	Records of occurrence in publications, n (%)
1	Humans	153 (100)
2	Female	127 (83.01)
3	Male	113 (73.86)
3	Students	113 (73.86)
5	Young Adult	99 (64.71)
6	Psychology	96 (62.75)
7	Universities	95 (62.09)
8	Adult	93 (60.78)
9	Epidemiology	86 (56.21)
10	Exercise	85 (55.56)

#### Leading authors, institutions, and countries/territories

Table 4 lists the researchers with the largest publications in the field over the past 20 years. Griffiths, MD (WoS, 9; Scopus, 6) and Rogowska, AM (WoS, 8; PubMed, 1; Scopus, 5) are undoubtedly the most leading researchers, ranking first and second in terms of publications, respectively, followed by Peltzer, K (WoS, 7; PubMed, 1; Scopus, 7) and Sikder, MT (WoS, 7; Scopus, 7). Additionally, three researchers from China Anhui Medical University (Tao, FB, Tao, SM, and Wu, XY) have also performed well, publishing 6 papers in the WoS dataset and five papers in the PubMed and Scopus datasets. It is worth noting that the listed researchers have different geographical backgrounds, with the majority being from China (9 researchers from 7 different universities), followed by Switzerland (4 researchers from the University of Basel). Two researchers each from the United States, Australia, and Poland, and other productive researchers belong to higher education institutions in the United Kingdom, South Africa, and Bangladesh.

**Table 4:** Leading authors in the field of exercise therapy of college students in depression.

Authors	Institution	Country	Publication count, n (%)			Citations	Average citation	H-index
			WoS (*n* = 1,085)	PubMed (*n* = 153)	Scopus (*n* = 696)			
Griffiths, MD	Nottingham Trent University	United Kingdom	9 (0.829)	__*	6 (0.86)	176	19.56	6
Rogowska, AM	Opole University	Poland	8 (0.737)	1 (0.654)	5 (0.72)	97	12.13	3
Peltzer, K	University of Limpopo	South Africa	7 (0.645)	1 (0.654)	7 (1.01)	251	35.86	6
Sikder, MT	Jahangirnagar University	Bangladesh	7 (0.645)	__	7 (1.01)	338	48.29	6
Chen, ST	Victoria University	Australia	6 (0.553)	__	6 (0.86)	37	6.17	2
Chen, WY	University of Michigan	United States	6 (0.553)	2 (1.307)	5 (0.72)	40	6.67	3
Gerber, M	University of Basel	Switzerland	6 (0.553)	3 (1.961)	__	230	38.33	6
Kusnierz, C	Opole University of Technology	Poland	6 (0.553)	__	3 (0.43)	88	14.67	2
Pengpid, S	Mahidol University	Thailand	6 (0.553)	1 (0.654)	7 (1.01)	213	35.5	5
Tao, FB	Anhui Medical University	China	6 (0.553)	5 (3.268)	5 (0.72)	159	26.5	4
Tao, SM	Anhui Medical University	China	6 (0.553)	5 (3.268)	5 (0.72)	159	26.5	4
Wu, XY	Anhui Medical University	China	6 (0.553)	5 (3.268)	5 (0.72)	159	26.5	4
Bartholomew, JB	University of Texas	United States	5 (0.461)	__	__	117	23.4	5
Brand, S	University of Basel	Switzerland	5 (0.461)	3 (1.961)	__	210	42	5
Hasan, MT	Monash University	Australia	5 (0.461)	__	4 (0.57)	173	34.6	3
He, ZH	Peking University	China	5 (0.461)	2 (1.307)	4 (0.57)	45	9	3
Holsboer-trachsler, E	University of Basel	Switzerland	5 (0.461)	3 (1.961)	__	210	42	5
Liu, Y	Shanghai Sport University	China	5 (0.461)	1 (0.654)	__	47	9.4	3
Meng, SQ	Yangzhou University	China	5 (0.461)	2 (1.307)	3 (0.43)	48	9.6	3
Puhse, U	University of Basel	Switzerland	5 (0.461)	3 (1.961)		210	42	5
Shi, L	BNU-HKBU United International College	China	5 (0.461)	2 (1.307)	3 (0.43)	59	11.8	3
Wang, K	University of Chinese Academy of Sciences	China	5 (0.461)	__	__	39	7.8	4
Yu, Q	Shenzhen University	China	5 (0.461)	__	3 (0.43)	49	9.8	3

*__: no related records.

Table 5 lists the leading research institutions in terms of publication output in the field. The University of Texas System (WoS, 23; PubMed, 3;) and State University System of Florida (WoS, 22; PubMed, 9; Scopus, 3) from the United States are far ahead in the field, followed by the University of California System (WoS, 19; PubMed, 2) and Harvard University (Wos, 17; PubMed, 1; Scopus, 7). Additionally, the University of London (WoS, 16; PubMed, 1; Scopus, 5) and Nottingham Trent University (WoS, 15; Scopus, 11) from the United Kingdom have also made significant contributions to the field.

**Table 5:** Leading Institutions in the field of exercise therapy of college students in depression.

Institution	Publication count, n (%)			Citations	Average Citations	H-index
	WoS (*n* = 1,085)	PubMed (*n* = 153)	Scopus (*n* = 696)			
University of Texas System	23 (2.12)	3 (1.96)	__	1,030	44.78	13
State University System of Florida	22 (2.03)	9 (5.88)	3 (0.43)	1,119	50.86	14
University of California System	19 (1.75)	2 (1.31)	__	602	31.68	9
Harvard University	17 (1.57)	1 (0.65)	7 (1.01)	1,370	81.06	10
University of North Carolina	17 (1.57)	6 (3.92)	3 (0.43)	334	19.65	8
University of London	16 (1.47)	1 (0.65)	5 (0.72)	720	45	11
Nottingham Trent University	15 (1.38)	––*	11 (1.58)	463	30.87	9
Pennsylvania Commonwealth System of Higher Education Pcshe	15 (1.38)	1 (0.65)	8 (1.15)	411	27.4	9
University of Michigan	14 (1.29)	2 (1.31)	6 (0.86)	400	28.57	7
University of Minnesota System	14 (1.29)	2 (1.31)	__	400	28.75	7
University of Toronto	14 (1.29)	__	9 (1.29)	195	13.93	8
University System of Ohio	14 (1.29)	1 (0.65)	3	603	43.07	10
University of Minnesota Twin Cities	13 (1.2)	2 (1.31)	8 (1.15)	919	70.69	12
Victoria University	13 (1.2)	1 (0.65)	8 (1.15)	172	13.23	5
Jahangirnagar University	12 (1.11)	__	13 (1.87)	448	37.33	9
Peking University	12 (1.11)	3 (1.96)	12 (1.72)	121	10.08	7
Shenzhen University	12 (1.11)	__	13 (1.87)	52	4.33	4
University of Florida	12 (1.11)	7 (4.58)	6 (0.86)	544	45.33	9
University of Melbourne	12 (1.11)	__	5 (0.72)	197	16.42	8
University System of Georgia	11 (1.01)	__	4 (0.57)	171	15.55	6

*__: no related records.

Table 6 lists the leading countries/territories in research on exercise therapy of college students in depression. The United States is undoubtedly in the absolute leading position in this field, recording 321 (29.59%), 58 (37.91%), and 192(27.59%) publications in WoS, PubMed, and Scopus respectively, all in first place. Following close behind is China, which has published 197 (18.06%), 23 (15.03%), and 175 (25.14) in the three datasets, respectively. The last three of the top five in the WoS dataset are occupied by three English-speaking countries or regions, namely Australia (85.7.83%), United Kingdom (85.7.83%), and Canada (64.5.90%). The remaining countries in the table are mainly distributed in Europe (Turkey, Germany, Spain, Poland, etc.), East Asia (South Korea, Japan), and South America (Brazil). This indicates that research on depression and physical activity among college students has received different degrees of attention around the world, especially in Europe.

**Table 6:** Leading countries/territories in the field of exercise therapy of college students in depression.

Rank	Countries/territories	Publication count, n (%)			Citations	Average citation	H-index
		WoS (*n* = 1,085)	PubMed (*n* = 153)	Scopus (*n* = 696)			
1	United States	321 (29.59)	58 (37.91)	192 (27.59)	9,771	30.44	51
2	China	196 (18.06)	23 (15.03)	175 (25.14)	2,581	13.17	25
3	Australia	85 (7.83)	7 (4.58)	38 (5.46)	1,271	14.95	20
3	United Kingdom	85 (7.83)	3 (1.96)	53	2,142	25.2	26
5	Canada	64 (5.90)	1 (0.65)	36 (5.17)	1,478	23.09	19
6	Turkey	52 (4.79)	4 (2.61)	33 (4.74)	543	10.44	12
7	Germany	43 (3.96)	4 (2.61)	29 (4.17)	1,327	30.86	18
8	Spain	41 (3.78)	13 (8.50)	27 (3.88)	699	17.05	12
9	Poland	34 (3.13)	3 (1.96)	13 (1.87)	323	9.5	8
10	South Korea	33 (3.04)	2 (1.31)	27 (3.88)	446	13.52	9
11	Saudi Arabia	26 (2.40)	2 (1.31)	15 (2.16)	171	6.58	7
12	Italy	25 (2.30)	7 (4.58)	22 (3.16)	338	13.52	10
13	Japan	24 (2.21)	2 (1.31)	16 (2.30)	264	11	10
14	Brazil	21(1.94)	4 (2.61)	17 (2.44)	279	13.29	8
15	France	19 (1.75)	4 (2.61)	14 (2.01)	501	26.37	9

### Analysis of scientific collaboration network

**Figure 3:**
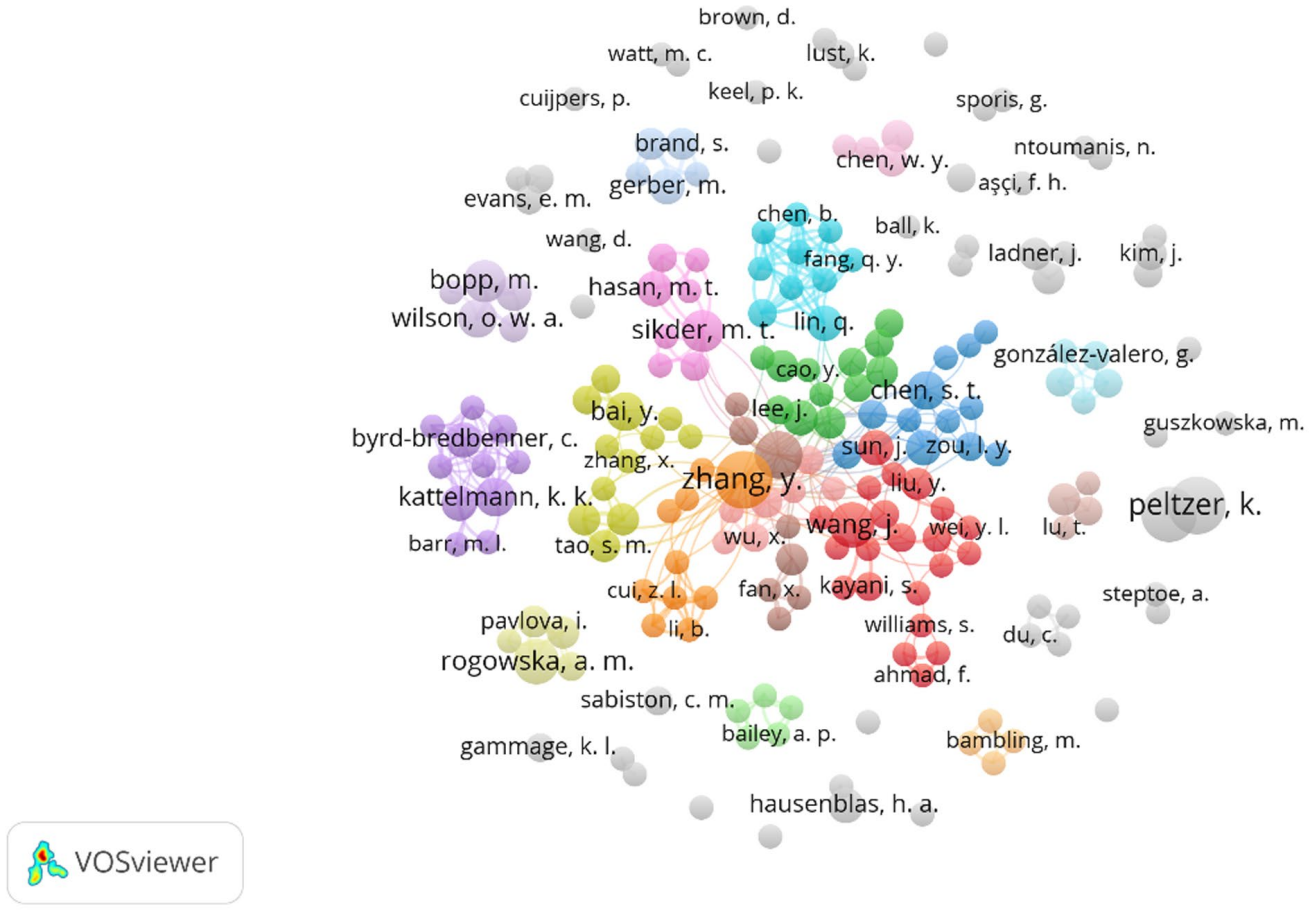
Scientific collaboration networks between authors. Only authors with three or more publications were considered.

In this study, we used VOSViewer to explore the potential scientific collaboration network in the field of exercise therapy of college students in depression over the past 20 years, including researchers and countries/territories. Figure 3 shows the collaboration network relationships among authors in the dataset. In this analysis, only researchers with at least 3 publications were considered, and a total of 201 authors out of the 5,651 researchers were included in the network construction. The map shows the presence of multiple productive collaboration networks in this field, contributing to the development of this field. Eight cross-regional collaborative networks at the center of the graph, with close ties and large numbers of researchers. Obviously, the collaborative relationship between Chinese researchers is significantly stronger than that of other countries in this field. For example, the largest collaborative network (red cluster) consists of 4 researchers from Canada and 18 researchers from China’s Wuhan, Beijing, Hangzhou, etc. The second largest collaborative network (green cluster) is composed of 3 researchers from the United States and 9 researchers from China’s Guangzhou, Shanghai and others. This indicates that Chinese researchers pay more attention to in the fields of exercise therapy of college students in depression, and to some extent, have formed close collaborative relationships with some foreign researchers. Interestingly, there are also some international research groups in this field. For example, the dark blue cluster is composed of 12 higher education research institution members from China, Australia, and the United Kingdom, while the brown cluster represents a more extensive international research team, including seven researchers from China, Bangladesh, the United Kingdom, and Hungary. The researchers in this cluster also have close collaborations with those in the violet cluster, orange cluster, dark green cluster, and dark blue cluster.

**Figure 4:**
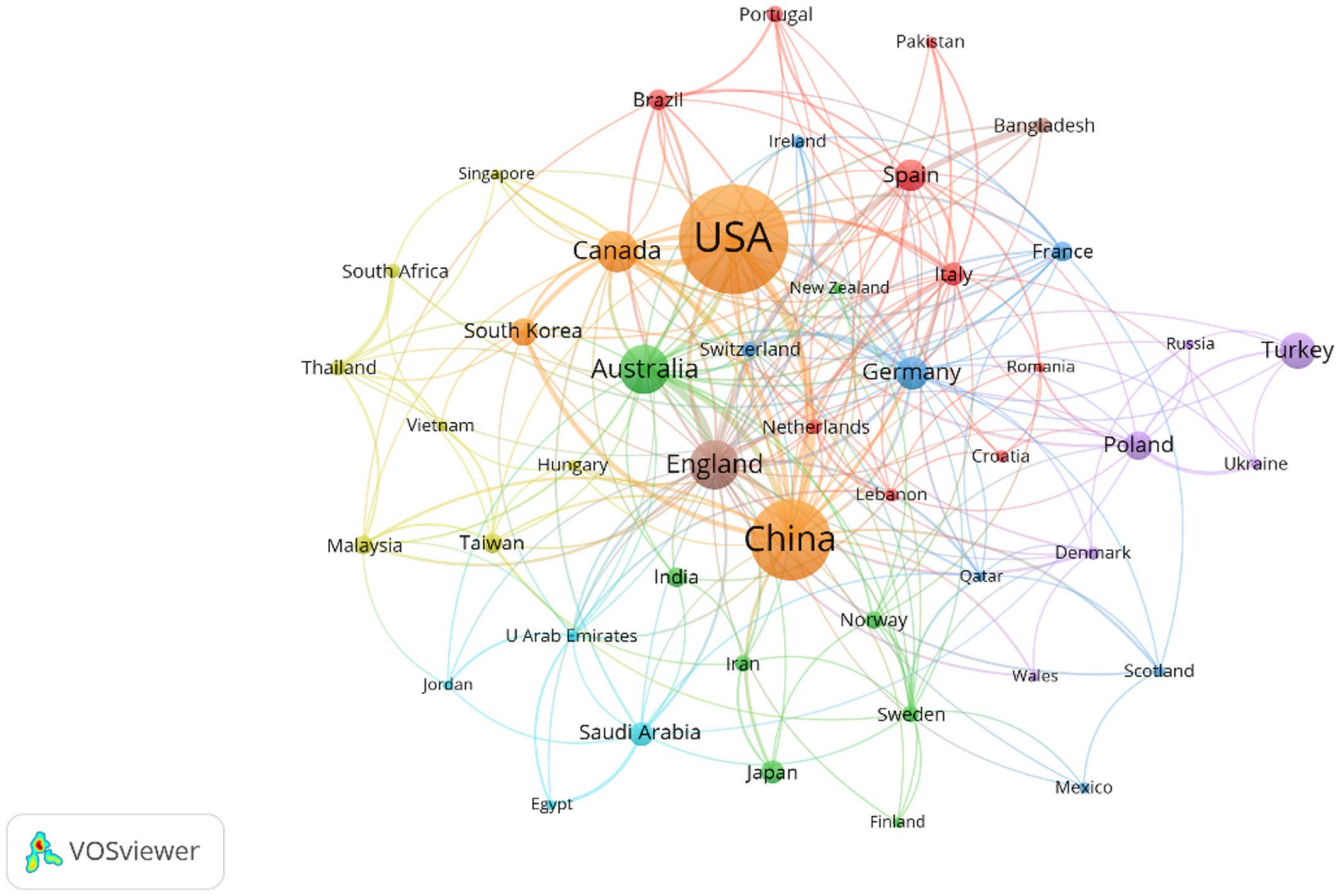
Scientific collaboration networks between countries/territories. Only countries with 6 or more publications were considered.

The cross-national collaborative network for research on exercise therapy of college students in depression over the past 20 years is shown in Figure 4. In this analysis, only scientific research collaborations between countries with 6 or more publications were considered, and 47 of the 91 countries were ultimately included in the network analysis. The United States occupies the center of the map, forming strong partnerships with other countries and regions, and it is clustered with China, Canada, and South Korea (Orange cluster). Overall, international collaboration in this field is heavily influenced by language, geography, and cultural background. For example, the largest cluster (red cluster) includes seven European countries (Brazil, Italy, Spain, etc.) and two Asian countries (Lebanon and Pakistan), who have similar cultural backgrounds and English as their common language. Additionally, six countries from Europe and four countries from the Arab countries form the purple cluster and light blue cluster, respectively. This indicates that, based on our data, current international collaborative research in this area is still relatively sparse and tends to be localized.

### Analysis of co-cited journals

**Figure 5:**
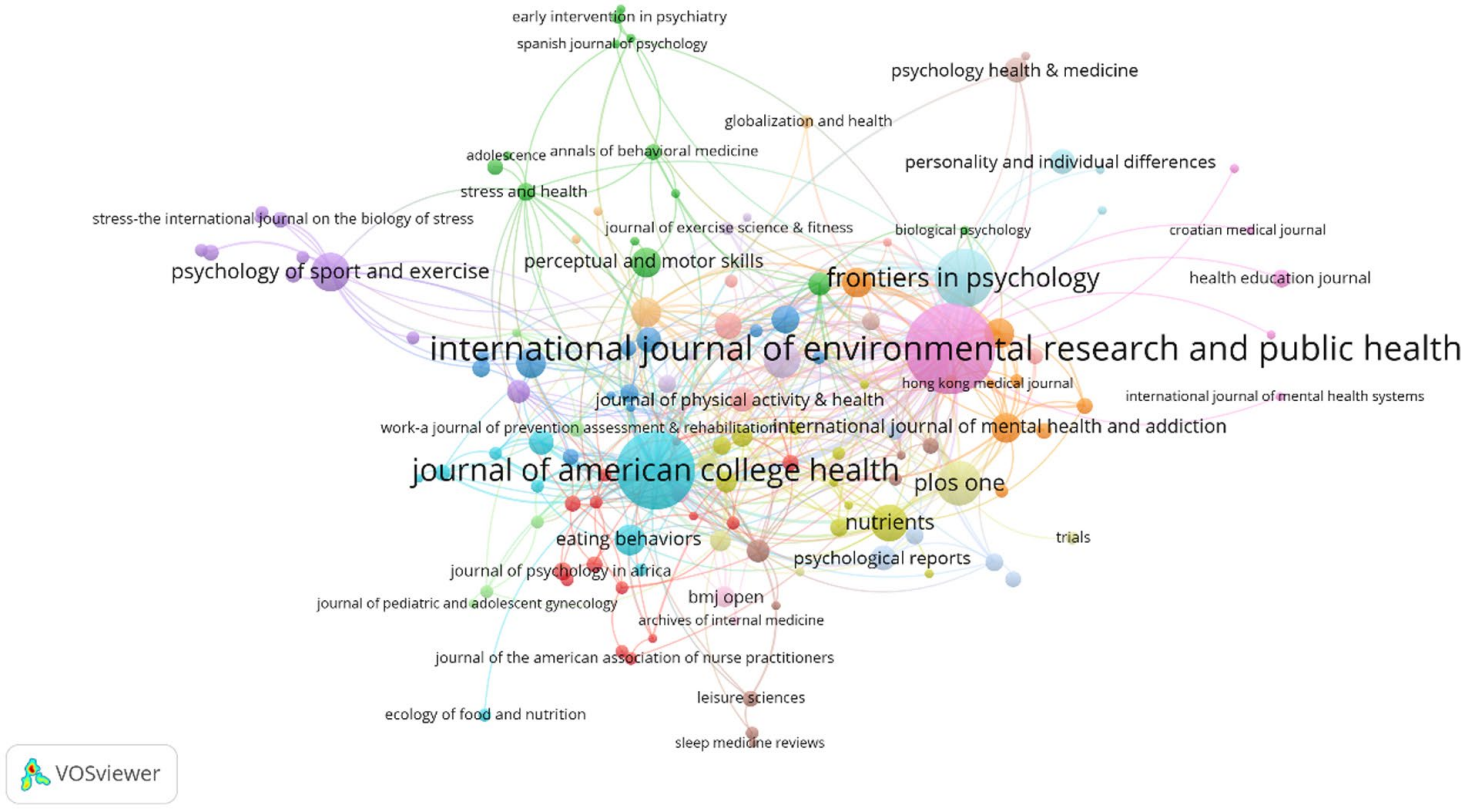
Map of clustered network journals based on co-citation data. Only countries with 25 or more publications were considered.

To explore the underlying discipline basis in the research fields of exercise therapy of college students in depression, a journal co-citation analysis of the literature in the dataset was performed by using VOSViewer. In this analysis, only journals with at least 25 citations were considered, out of the total of 507 journals, 132 were included in the analysis. The nodes in the map represent journals, with their size reflecting the number of co-citations with other journals. The colors represent journal clusters, and journals within a cluster have higher co-citation relationships and stronger disciplinary connections. As seen from the Figure 5, the *International Journal of Environmental Research and Public Health* is the largest node, indicating the highest frequency and strongest relationship of citations, and belongs to the purple pink cluster, which also includes *Asian Pacific Journal of Cancer Prevention*, *Croatian Medical Journal*, *Health Education Journal*, *International Journal of Mental Health Systems* and *International Journal of Psychophysiology*. This group can be defined as a cluster of disciplines related to public health, medicine, health education and psychology. Overall, this study indicates that there is a certain degree of interdisciplinary in the research field of exercise therapy of college students in depression. For example, in the bottom left of the map, the light blue cluster represented by *Journal of American College Health* integrates journals related to behavioral science, cognitive science, nutrition, higher education, and psychology; in the upper left of the map, the purple cluster represented by *Psychology of Sport and Exercise*, consists of journals related to sports exercise, health education, neurosciences, and psychology; in the upper right of the map, the cyan cluster represented by *Frontiers in Psychology*, represents research and contributions of journals related to individuals, urban environment, and psychology to the development of this field; in the bottom right of the map, the lemon-yellow cluster represented by *PLoS One,* covers journals related to adolescent health and the sociology of sport. In short, many other interdisciplinary clusters still exist in elsewhere in the map, such as the green cluster, which collects journals related to adolescents, behavioral medicine, sports medicine, and health psychology, and the orange cluster, which includes journals related to psychiatry, addiction medicine, and sleep fields.

### Analysis of co-occurring keywords and visualization based on time

**Figure 6:**
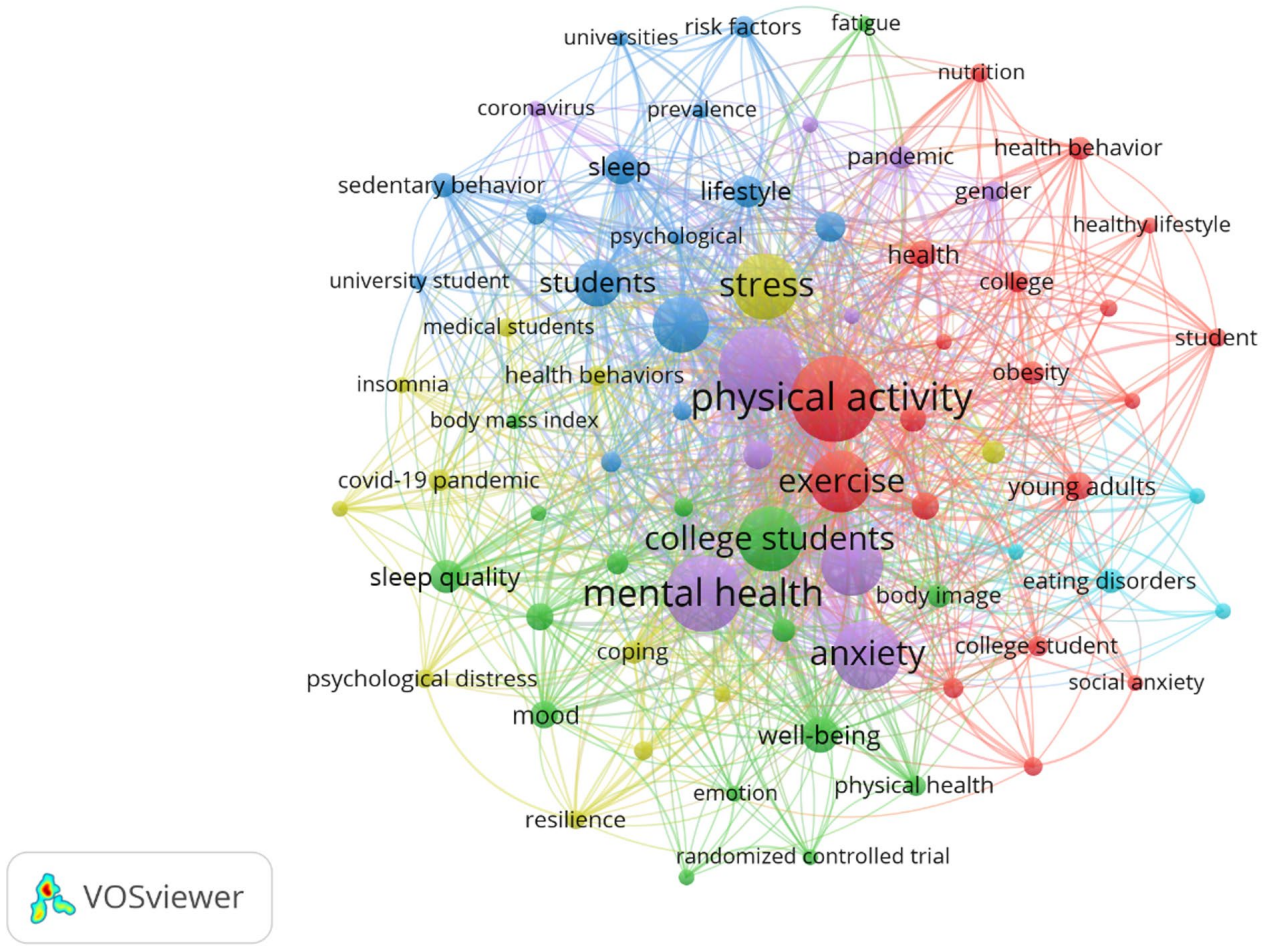
Co-occurrence network map of keywords. Only keywords with 8 or more co-citation frequency were displayed.

From January 2002 to December 2022, research hotspots in the field of exercise therapy of college students in depression are shown in Figure 6. VOSViewer provides us with a visual expression by co-occurrence analysis of keywords from all publications in the dataset. Co-occurrence of keywords refers to the frequency of two subject words appearing in the same article, aimed at indicating the relationship between the two subjects. In this analysis, we only consider keywords that appear 8 times or more, and out of the 2011 keywords, 75 keywords were included in this analysis. Keywords are the core subjects of articles, and the frequency of keyword appearance represents the popularity of topic research. The larger the node of keywords with higher appearance frequency, and the thickness of the lines represents the co-occurrence strength between different keywords. The top five keywords with the highest frequency in the dataset are: physical activity (frequency: 219), depression (frequency: 195), mental health (frequency: 175), anxiety (frequency: 142), and stress (frequency: 129), which suggests that common mental health issues faced by college students in higher education systems include depression, anxiety, and stress, and behaviors that contribute to mental health, such as physical activity, are receiving more and more attention from scholars. In addition, the impact of COVID-19 (frequency: 117), sleep (frequency: 36), and diet (frequency: 25) on the emotions of college students has also attracted the attention of researchers, indicating that, to some extent, behaviors related to health such as the pandemic, sleep and diet also affect college students’ physical activity and mental health, which coincides with the research of Zhou et al. (2022). The results of the cluster analysis suggest that the six general themes seemed to summarize the topic foci of in the research field of exercise therapy of college students in depression over the past 20 years. First, researchers are generally interested in the health-promoting factors of college students, and high-frequency keywords such as health behavior, health lifestyle, nutrition, diet, exercise and physical activity (red cluster) often appear in the map. Second, the impact of students’ body image on emotions is another widely discussed topic, especially social physique anxiety, body mass index, fatigue, body image, mood, and wellbeing (green cluster). Lack of sports exercise often leads to weight gain in students and has a negative impact on their mental health (Soysal et al., 2023). The third topical area in this field is negative behaviors that affect the mental health of college students, such as risk factors, sedentary behavior, prevalence, sleep, and psychological (blue cluster). The other three popular topics shown in the map include stress-increasing factors, such as stress, insomnia, loneliness, COVID-19 pandemic, and psychological distress (yellow cluster); depression and its coping measures, such as depression, anxiety, meditation, mindfulness, and mental health (purple cluster); and diet problems, such as eating disorders (light blue cluster).

**Figure 7:**
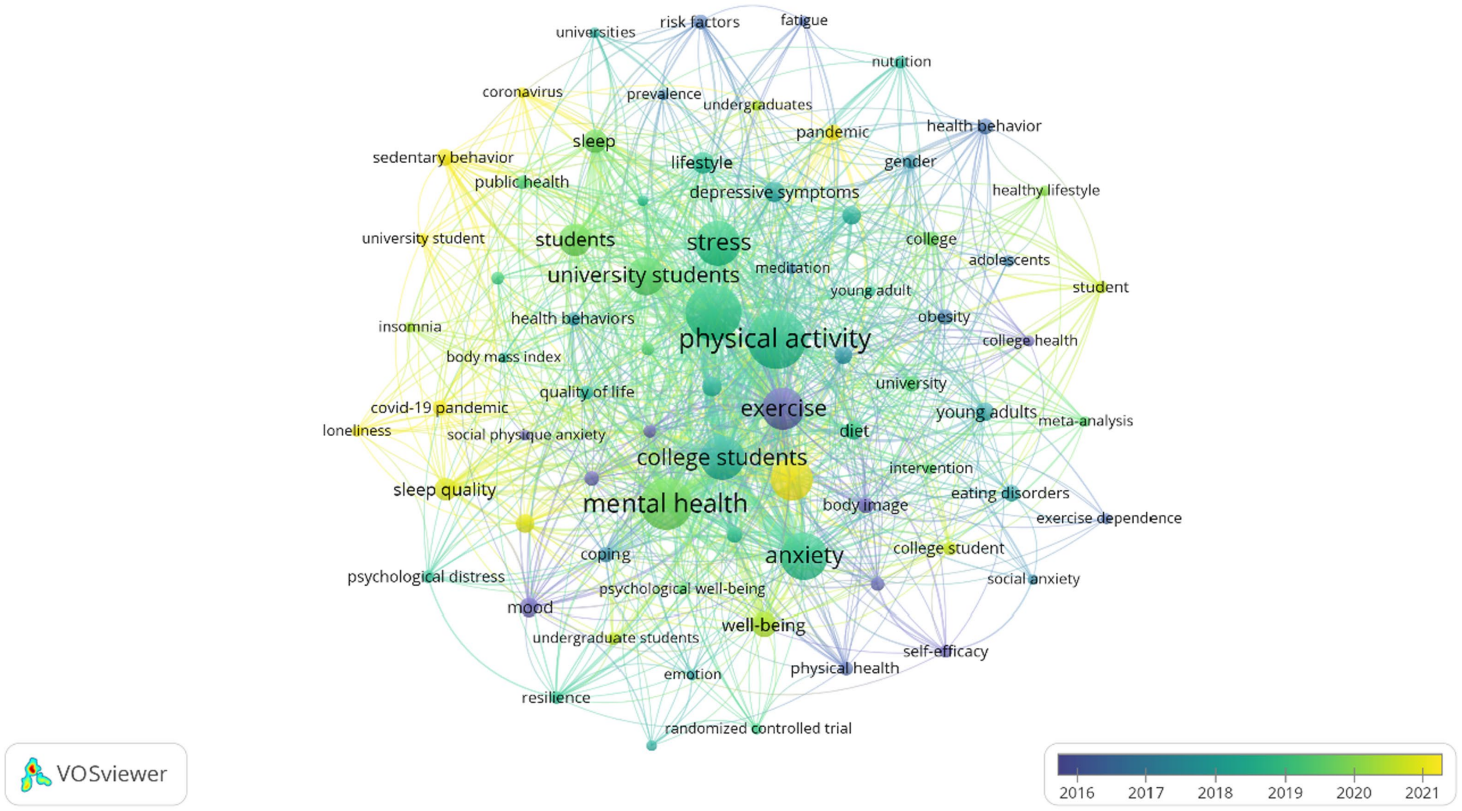
Average year map of keywords used from 2002 to 2022.

To show the trend network map of research topics in this field from 2002 to 2022, a time-based visual analysis of keywords was performed, as shown in Figure 7. Dark blue represents the earlier average year of appearance of the keywords, and the yellow represents the later average year of appearance. The size of the nodes represents the level of research popularity, and the legend shows the average year. Around 2016, the hotspots of research in this field are mainly on the influence of health behaviors, body image and self-esteem on the group emotions of college students. The highly discussed research hotspots were around 2019, focusing on the impact of sports exercise, sleep, lifestyle on the symptoms of depression, stress levels, and mental health of college students. By around 2021, the impact of the pandemic and loneliness on the mental health and happiness of college students also sparked the interest of researchers.

## Discussion

This study used three citation databases, WoS, PubMed, and Scopus, to conduct a bibliometric analysis of publications in the field of exercise therapy of college students in depression from 2002 to 2022. The visualized maps and tables help us to display the research status, hotspots, and frontiers of this field more intuitively and clearly. The findings are as follows: the number of publications in this field has gradually increased over the past 20 years, indicating that researchers have made increased efforts to publish articles related to exercise therapy of college students in depression. The existing articles are mainly published in journals related to public health, education, psychiatry, or psychology, and the two journals with the most publications in three databases are *International Journal of Environmental Research and Public Health* and *Journal of American College Health*. In the past 20 years, the popular research fields and themes related to exercise therapy of college students in depression are mainly related to public health, psychology, psychiatry, and sports science in the WoS dataset, and the MeSH themes with the highest popularity in the PubMed dataset are mainly focused on young people, psychology, and exercise. Furthermore, Professor Mark D. Griffiths from Nottingham Trent University and Professor Aleksandra M Rogowska from Opole University are undoubtedly the most productive authors in this field, ranking first and second in terms of publications, respectively. For research institutions, the three higher education institutions from the United State, University of Texas System, State University System of Florida and University of California System, are the most dynamic institutions in this field, which makes United State the country with the greatest influence and the largest number of publications in this field. Science collaboration network analysis results show that in the past 20 years, collaboration among different authors in the field has mainly focused on domestic, especially in China. Although international research groups exist, they are small in scale and quantity, and collaboration between countries is often influenced by language, geographic location, and cultural background. In addition, journal co-citation analysis shows that research on exercise therapy of college students in depression is interdisciplinary to a certain extent, mainly including behavioral science, public health, and psychology. Currently, the hot topics of research in this field mainly focus on the relationship between college student depression and health behaviors, body image, and negative factors that affect college students’ mental health. Further research found that in recent years, research in this field mainly focuses on the impact of physical exercise, sleep, and lifestyle on the symptoms of depression, stress levels, and mental health of college students. Research on loneliness and the impact of the pandemic on college students’ mental health and wellbeing is also slowly increasing. Finally, we hope that future research will focus more on conducting clinical trials to find effective interventions for reducing the symptoms of college student depression. Furthermore, the complex relationship between the impact of exercise on college student depression and other health-related behaviors will be further validated through more rigorous research.

### Collaboration needs to be strengthened

Cross-border collaboration has become a mainstay of knowledge production in multiple scientific fields and is widely promoted as a means to cultivate research quality, improve resource utilization and influence (Hsiehchen et al., 2015). Despite substantial contributions to research in the areas of exercise therapy of college students in depression in many countries and regions over the past two decades, the distribution of research contributions has been highly uneven. Except for China, Turkey, and Poland, the core producing countries in this field are developed countries, with the top five research institutions all located in the United States. Developing countries seem to contribute only a small fraction of publications in this field, and underdeveloped regions contribute even less. The gap in research capacity between underdeveloped countries and developed countries and its impact on global health research trends can no longer be ignored (Akinremi, 2011). We hope that developed countries will engage in more collaboration with underdeveloped countries in this field and recommend that developing countries conduct more research and collaborate with other countries or institutions. In the network of co-author collaborations, authors form collaboration networks with different densities, but most networks are limited to small groups with few connections between groups, indicating that the research groups were relatively dispersed. Moreover, although there are a few transnational collaborations within the map, they seem to be limited to researchers from neighboring countries or the same region, and are heavily influenced by language, geographic location, and historical culture. Additionally, cultural similarity, quality of collaboration institutions and prosocial norms and the increasing cost of collaboration also complicate the formation and development of research partnerships (Dorrough et al., 2022). Limited collaboration within and between countries poses a challenge to the development of a field. Therefore, breaking boundaries in methods and techniques, meeting people from different cultures and regions, immersing oneself in these cultures, and sharing information, resources, and skills can promote better development of a field through the aggregation of talent, technology, and other resources (Rolfe et al., 2004; Bouras et al., 2018). Therefore, it is necessary to expand collaboration between authors and countries in order to carry out research more deeply and produce high-quality publications in the future. In this way, relevant research will become more globally applicable.

### The discipline foundations in exercise therapy of college students in depression field

In this study, we found that research on the exercise therapy of college students in depression was mainly published in psychology and public health journals, and the research fields were mainly focused on public health, psychology, psychiatry, and sports science. The results of the co-citation analysis of the journals indicated that, to some extent, research on exercise therapy of college students in depression is interdisciplinary, and behavioral science, public health, and psychology were identified as core research subjects in this field. This suggests that a comprehensive or interdisciplinary approach is often required in this field of research to better understand each research topic, which is similar to the viewpoint of Stewart-Brown (2015). Moreover, nutrition journals (*Eating Behaviors* and *Nutrients*) occupy a middle-lower position in Figure 7 and are closely linked with other journals. Diet and obesity can directly influence emotions, and stress-related mental disorders may lead to changes in dietary habits. Unhealthy eating patterns can result in low levels of physical activity, which often have negative impacts on students’ mental health (Bremner et al., 2020; Kuipers et al., 2021). Additionally, Sanna et al. (2021) study suggests that olfactory identification impairment, decreased attention, and language decline are associated with an increase in depression severity. These indicates that nutrition is a potential discipline for promoting development in this field. Additionally, journals from internal medicine and sleep medicine (*Archives of Internal Medicine* and *Sleep Medicine Reviews*) have also made a certain contribution to this field. Many chronic diseases, including cardiovascular disease, metabolic disorders, and inflammatory diseases, are one of the most common comorbidities of depression. The symptoms or treatment of internal medicine diseases may interfere with the treatment of depression, and conversely, the symptoms of depression may reduce adherence to treatment for these diseases (Ghrouz et al., 2020). Persistent sleep disorders are one of the most common symptoms of MDD in adolescents, and persistent insomnia can increase the risk of developing depression by more than two times (Reynolds et al., 2020; Zhang M.M. et al., 2022). Another meta-analysis found that moderate to high-intensity physical activity produces better sleep quality, thus improving students’ levels of mental health and alleviating symptoms of depression (Memon et al., 2021). In general, these results indicate that in the field of exercise therapy of college students in depression, it is a development pattern of multiple disciplines coexisting and coordinating with each other, mainly consisting of behavioral science, public health, and psychology, with nutrition, internal and sleep medicine, and other health-related disciplines as supplementary.

### The research hot spots in exercise therapy of college students in depression field

Keywords are highly concise summaries of the literature. In this study, we conducted co-occurrence, clustering, and time-based visual analysis of keywords, and summarized three research hotspots in the fields of exercise therapy of college students in depression.

First, depression prevalence among college students is high and varies among individuals. Through co-occurrence analysis of keywords we found that four of the top five keywords were related to depression indicating that psychological problems related to depression have been a research hotspot in this field over the past two decades. MDD is a common disease in all countries that conduct epidemiological surveys yet the proportion of treated depression patients is still low in many parts of the world (Kessler and Bromet, 2013). WMH surveys show that treatment rates are even low in many other developed countries and remain consistently low in developing countries (Wang et al., 2007). Depression is a serious mental health issue that is often overlooked and untreated in the college students with bad consequences. Recently a survey conducted in Jordan (Naser et al., 2021) found that in a sample of 1,582 undergraduates the prevalence of depression was 22.3% and the prevalence of anxiety was 15.8% which indicated that the mental health problems of college students could not be ignored. Interestingly we found that research in the past decade has been more inclined to focus on the prevalence of depression among different groups such as gender or race. A study from the United States showed that the level of depression among Asian Americans is significantly higher than that of white people and the proportion of depression in women is higher than that in men (Young et al., 2010). Similarly a cross-national study from 23 European countries showed that the prevalence of depression in women is approximately twice that in men (Van de Velde et al., 2010). With the progress of research more studies on the prevalence of depression among different groups of college students will be conducted in the future. Therefore we can have a more accurate understanding of the depression problems of different populations and based on this targeted intervention measures can be designed for different groups in the future.

Second, the interaction between the level of physical activity and the degree of depression among college students. In the co-occurrence analysis of keywords, physical activity ranked first with a frequency of 219. In addition, in the clustering analysis, physical activity was included in the red cluster as a health-promoting factor. More publications have investigated the relationship between physical activity and mental health, including depression, anxiety, and other mental disorders, and they interact with each other through complex bilateral relationships. Cahuas et al. investigated the relationship between physical activity and depression among college students. The results indicated that rigorous physical activity may help reduce and regulate depressive symptoms in college students, but this effect may vary by gender, as there was no significant relationship between physical activity levels and depression in females (Cahuas et al., 2020). A systematic review containing 59 studies found that lower levels of physical activity were associated with higher levels of depressive symptoms in patients with depression (Vancampfort et al., 2015). However, most studies are cross-sectional and cannot prove causality between physical activity and depression. Recently, some longitudinal studies have confirmed that appropriate physical activity can effectively alleviate depressive symptoms, and there is a bidirectional predictive relationship between them (Hellem et al., 2020; Jo et al., 2021). However, this is not enough, and more research is needed in the future to supplement and support the current research findings. In future longitudinal studies, large-scale studies should be conducted to verify the complex mechanism between physical activity and depression. Furthermore, it has been reported that college students engage in less physical activity, and physical activity levels are lower among higher-grade students, which can lead to poor health outcomes (Garcia Puello et al., 2015; Soto-Ruiz et al., 2019). In summary, the cross-sectional and longitudinal evidence regarding the relationship between physical activity and depression remains equivocal. Therefore, we suggest that researchers should consider physical activity and depression as interdependent behaviors and conduct high-quality research among different cohorts of college students to enrich future conclusions.

Third, body image, sleep, and diet affect college students’ physical activity levels and depression. In the cluster analysis of keywords, we found that in the field of exercise therapy of college students in depression in the past two decades, factors such as body image, sleep and diet are also worthy of attention. A survey from Chinese college students found that most students were dissatisfied with their body image, with a similar proportion of male and female, and among female participants, dissatisfaction with body image is negatively correlated with self-esteem and subjective wellbeing, and positively correlated with negative emotions (Wang et al., 2018). Sleep is an important physiological function of the human body. Good sleep can alleviate fatigue and restore vitality. Healthy sleep plays an important role in maintaining brain function and cognition (Tarokh et al., 2016). A cross-sectional study from India demonstrated a significant correlation between sleep quality, physical activity level, and psychological wellbeing among college students (Ghrouz et al., 2019). Poor eating habits tend to increase the prevalence of overweight and obesity. Recent research has shown that the detection rate of various mild mental health problems in obese college students is as high as 44.56%, and the detection rate of various moderate mental health problems is 9.69%. Compared with the national youth norms, obese college students are prone to psychological problems such as somatization, obsessive–compulsive symptoms, anxiety, terror, psychosis, hostility, and paranoia (Wu, 2022). In addition, low-levels of exercise are associated with overweight and obesity, and appropriate exercise and good dietary habits can play a positive role in reducing the incidence of obesity (Maatoug et al., 2013; Rangel Caballero et al., 2014). These studies suggest that there is a certain correlation between the body image, sleep quality, and dietary habits of college students and depression and exercise, but their underlying mechanisms remain unclear. As the research deepens, more indirect relationships will be explored to expand current findings. If possible, scholars or clinical experts can apply relevant theories to intervene in intermediate or moderating factors to help clarify the mechanisms and we look forward to credible research results that will help us understand the complex mechanisms involved and reduce the incidence of depression in students, and improve their mental health.

Fourth, potential mechanisms of exercise intervention in depression. The study of the mechanisms underlying the therapeutic effects of exercise for depression has attracted increasing academic attention in recent years. A clinical trial demonstrated that exercise could improve patients’ cardiopulmonary function and increase the cortical thickness of key region sensitive to depression in the brain to alleviate depressive symptoms (Gujral et al., 2019). Among patients with depression, a reduction in the volumes of the hippocampus, anterior cingulate cortex, prefrontal cortex, striatum, and amygdala, as well as compromised white matter integrity, have been frequently observed, which may represent potential targets for exercise treatment for depression (Gujral et al., 2017). Furthermore, Xie et al. (2021) systematic review revealed that the mechanisms underlying exercise treatment for depression are related to both psychological and physiological factors, including improvement in patients’ psychological status and cognitive ability, anti-inflammatory and antioxidant effects, and reduction of neuroinflammation levels. Moreover, Lee and Baek (2017). review indicated that in psychiatric disorders such as depression, exercise can protect brain function by increasing neurogenesis through activation of the Wnt signaling pathway. There is growing evidence suggest that the gut microbiota plays a significant role in the development of depression (Koopman et al., 2017; Taylor and Holscher, 2020). Interestingly, exercise can regulate the gut microbiota and alleviate toxin-induced gastrointestinal syndrome. The gut microbiota can also promote the beneficial effects of exercise on cognition and emotion, either directly through serotonin signaling or indirectly through the regulation of metabolism and exercise performance (Yuan et al., 2015). Briefly, while much progress has been made in the study of the mechanisms underlying exercise treatment for depression, further research is still needed.

## Strengths and limitations

The highlight of this study is that it is the first bibliometric analysis in the field of exercise therapy of college students in depression over the past 20 years. Multiple vivid knowledge maps and tables were used to illustrate the development trajectory of this field, identify the core producers, reveal the scientific collaboration networks among authors and countries, and clarify the interdisciplinary nature of research in this field. The study also analyzed and discussed the hotspots of this field, providing insights for future research directions and methods. However, there are also some limitations in this study. First, publications were only retrieved from the WoS, PubMed, and Scopus databases, which limited the generalizability of the findings. Second, only journal articles and reviews were retrieved for analysis, excluding other relevant publications in the field such as book chapters and conference proceedings. Third, we only collected English-language literature retrieved from the three databases over the past 20 years through specific search strings, so we did not include all literature in the field, which may have affected the results summary. If possible, a more comprehensive search string should be designed for the prospective bibliometric analysis of the field to include more literature without being limited by language, document type, publication date, or database. Nevertheless, we believe that the bibliometric approach used in this study provides new insights into the development and current status of the field, as well as some challenges that hinder its progress.

## Conclusion

Overall, the results of this study shed light the trend of the field over the past 20 years and hold promise for a better understanding of the scientific patterns between depression and exercise among international university students. Firstly, researchers’ interest in exercise therapy of college students in depression has increased over the past 20 years and has continued in recent years, indicating that this field has not yet reached maturity and will continue to develop in the future. Secondly, the research in this field is relatively interdisciplinary, involving the fusion of several disciplines such as behavioral science, public health, psychology. We hope that in future research, researchers from different fields can participate in the study of exercise therapy of college students in depression, working together to promote the mental health of university students worldwide and reduce the incidence of depression. Thirdly, most of the research in this field is conducted by scholars from developed countries, and collaboration among scholars is often influenced by language, cultural background, and geographical differences. Therefore, it is necessary to break down the barriers between countries, expand collaboration among researchers and nations, and promote global research in this field. Fourthly, over the past 20 years, researchers have conducted in-depth research in multiple areas related to exercise therapy of college students in depression, and have achieved certain results. However, the evidence for research into the complex mechanisms between exercise and depression is insufficient, and the relationship between depression in university students and exercise with other health-related factors remains unclear. Therefore, in-depth longitudinal studies and expanded randomized controlled trials are necessary to consolidate the current research results. In summary, our study provides guidance and reference for researchers to contemplate and determine research directions, reduces the effort in exploring the boundaries of the field, and provides a theoretical foundation for further research in this area.

## Data availability statement

The raw data supporting the conclusions of this article will be made available by the authors, without undue reservation.

## Author contributions

ZA, SZ, ZZA, and DY: study design. DY and YM: data collection, analysis and interpretation. ZZA and DY: critical revision of the manuscript and drafting the manuscript. All authors: approval of the final version for publication.

## Funding

This study was supported by the Scientific Research Project of Hubei Provincial Department of Education in 2022 (Q20222014).

## Conflict of interest

The authors declare that the research was conducted in the absence of any commercial or financial relationships that could be construed as a potential conflict of interest.

## Publisher’s note

All claims expressed in this article are solely those of the authors and do not necessarily represent those of their affiliated organizations, or those of the publisher, the editors and the reviewers. Any product that may be evaluated in this article, or claim that may be made by its manufacturer, is not guaranteed or endorsed by the publisher.
